# Uveitis–glaucoma–hyphema syndrome with sclera-fixed posterior-chamber two-haptic intraocular lens in a highly myopic eye: a case report

**DOI:** 10.1186/s12886-020-1309-5

**Published:** 2020-01-10

**Authors:** Yu Du, Xiangjia Zhu, Jin Yang, Yinglei Zhang, Lei Cai, Yi Lu

**Affiliations:** 1 Department of Ophthalmology, Eye and Ear, Nose, and Throat Hospital, Fudan University, Shanghai, 200031 China; 2 Key Laboratory of Myopia, Ministry of Health, Shanghai, 200031 China; 3 Key Laboratory of Visual Impairment and Restoration, Shanghai, 200031 China

**Keywords:** Uveitis, Glaucoma, Hyphema syndrome, sclera-fixed intraocular lens, highly myopic eye

## Abstract

**Background:**

We report a case of uveitis–glaucoma–hyphema (UGH) syndrome in a highly myopic pseudophakic eye with seemingly normal positioning of a two-haptic intraocular lens (IOL).

**Case presentation:**

The patient was a 61-year-old woman suffering recurrent episodes of blurred vision, floaters, redness, elevated intraocular pressure (IOP), and pain in the right eye following implantation of a sclera-fixed IOL. The symptoms were alleviated by the systemic and topical administration of IOP-lowering and anti-inflammatory medications. A slit-lamp examination revealed depigmentation and atrophy of the iris, and a quiet anterior chamber in the right eye. Endophthalmitis caused by hypovirulent bacteria and UGH syndrome were both considered. Ultrasound biomicroscopy (UBM) and gonioscopy provided direct evidence of malpositioned IOL haptics, which pushed the root of the iris forward, resulting in persistent mechanical chaffing, the probable cause of UGH syndrome. IOL explantation resolved her symptoms. Negative bacterial culture results for the IOL excluded the possibility of endophthalmitis.

**Conclusions:**

Heightened awareness of underlying UGH syndrome and prompt UBM are important when doctors encounter a patient with a sclera-fixed IOL suffering from recurrent anterior segment inflammation and elevated IOP.

## Background

Uveitis–glaucoma–hyphema (UGH) syndrome is a triad characterized by recurrent episodes of anterior-chamber inflammation, increased intraocular pressure (IOP), microhyphema, and blurred vision. It is generally associated with contact between a malpositioned anterior-chamber intraocular lens (IOL) and the iris or ciliary body, leading to mechanical tissue trauma. In recent decades, the landscape of cataract surgery has seen the emergence of new placement techniques for IOLs. Currently, the overwhelming majority of IOLs are implanted in the capsular bag, minimizing the possibility of the IOLs contacting the uvea and reducing the incidence of UGH syndrome. Therefore, UGH syndrome is generally rare and is difficult for doctors to diagnose in the first place. In most cases, UGH syndrome can be treated medically, although the removal of the IOL is sometimes necessary [1]. Here, we present a rare case of UGH syndrome in a highly myopic eye with a sclera-fixed posterior-chamber two-haptic IOL.

## Case presentation

A 61-year-old woman who had suffered intermittent blurred vision, floaters, redness, and pain in the right eye for over 4 months presented at the Department of Ophthalmology, Eye & Ear, Nose, and Throat Hospital of Fudan University, Shanghai, China in September 2017. There was no previous history of glaucoma. The patient reported that she had undergone uneventful phacoemulsification with implantation of a posterior-chamber in-the-bag two-haptic IOL in her right eye in May 2003, 14 years before presentation. She had also undergone epiretinal membrane surgery in the same eye in June 2013. Both surgeries were followed by an uncomplicated early postoperative period. Four years after epiretinal membrane surgery (May 2017), the patient experienced sudden vision impairment, which was managed at her local hospital. The diagnosis was displacement of the IOL into the vitreous cavity. IOL explantation with suture scleral fixation of the original IOL was performed. One month later, the patient complained of blurred vision, floaters, redness, and pain in the right eye, and she was admitted to the local hospital. The patient’s visual acuity was CF/15 cm, and her IOP was 40 mmHg in the right eye, whereas the left eye was normal. She was administered systemic and topical IOP-lowering medication combined with an anti-inflammatory treatment. Her IOP had dropped to 17 mmHg and her visual acuity had improved to 0.6. However, in the following 4 months, the patient twice experienced sudden vision impairment with eye pain, redness, and floaters.

**Fig. 1:**
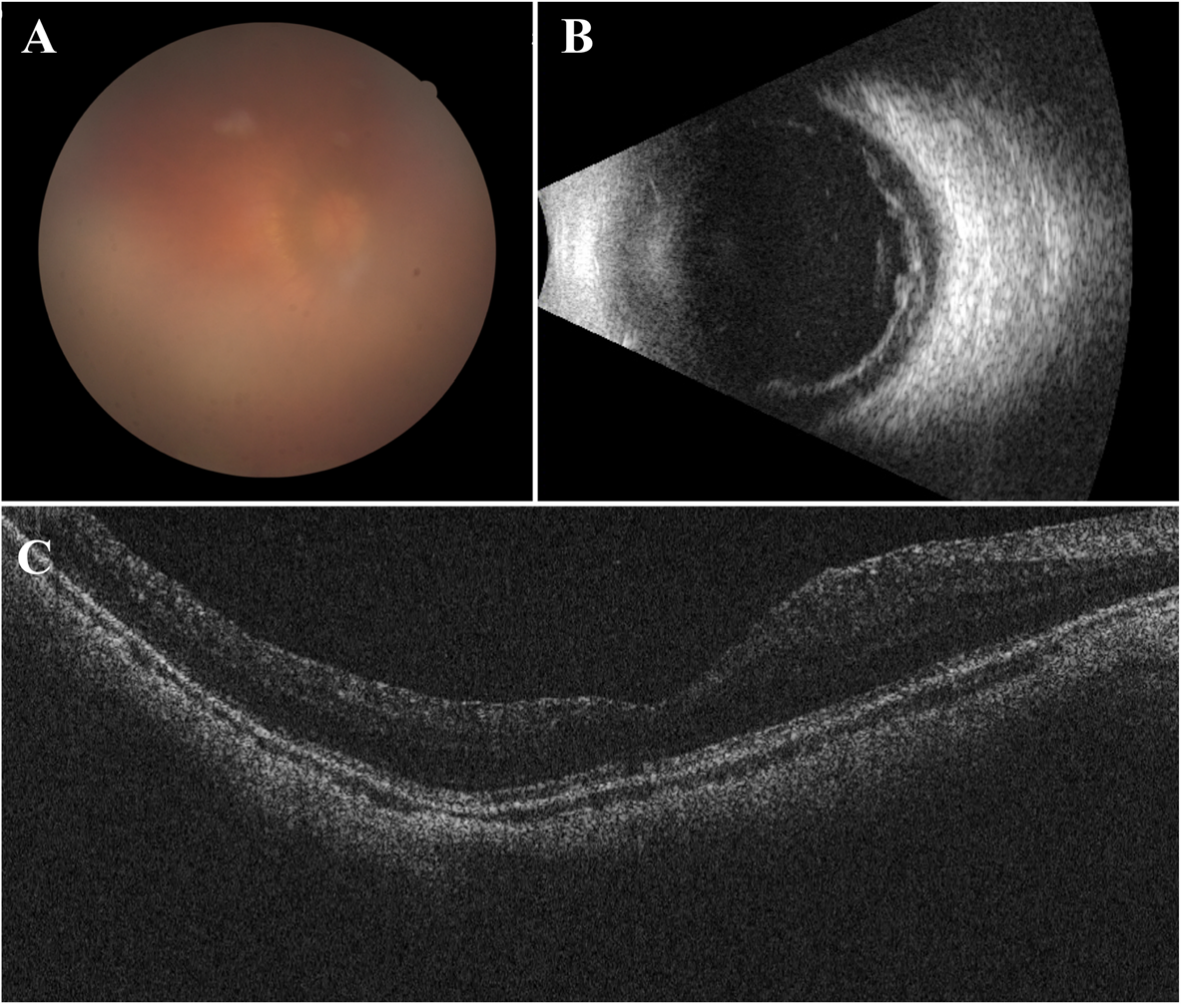
Ophthalmic examinations of the patient at presentation. **a** Fundus photography showed an opaque image of the posterior segment. **b** B-scan ultrasound showed a mass of echoes in the whole vitreous cavity and strip-like echoes in the peripheral region. **c** Optical coherence tomography showed a retinal structure that is common in highly myopic eyes

**Fig. 2:**
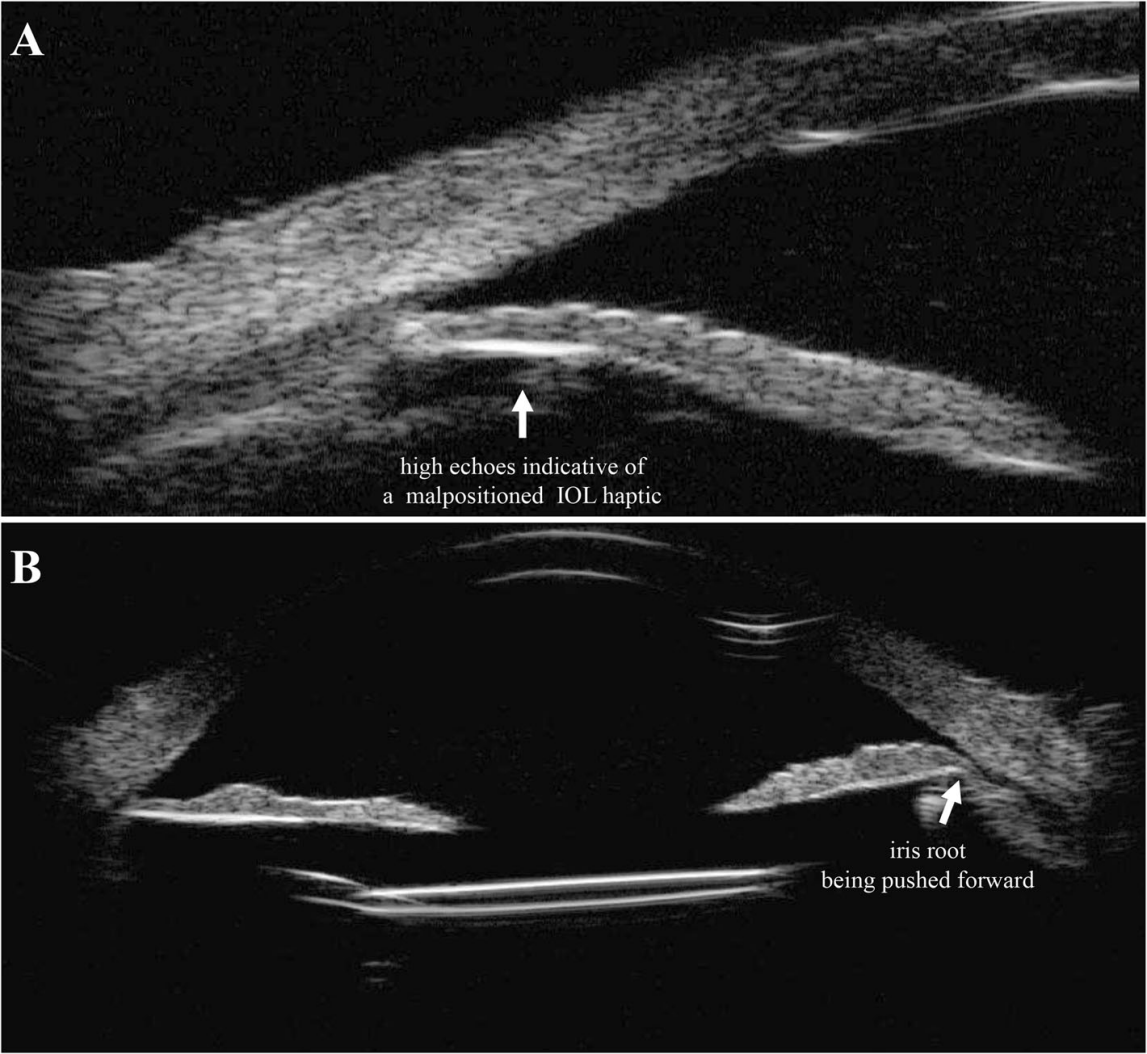
Ultrasound biomicroscopy. High echoes were detected in the sulcus at the 5 and 11 o’clock positions (2**a**, white arrow), indicating that the iris root was pushed forward by the malpositioned intraocular lens (IOL) haptic (2**b**, white arrow)

**Fig. 3:**
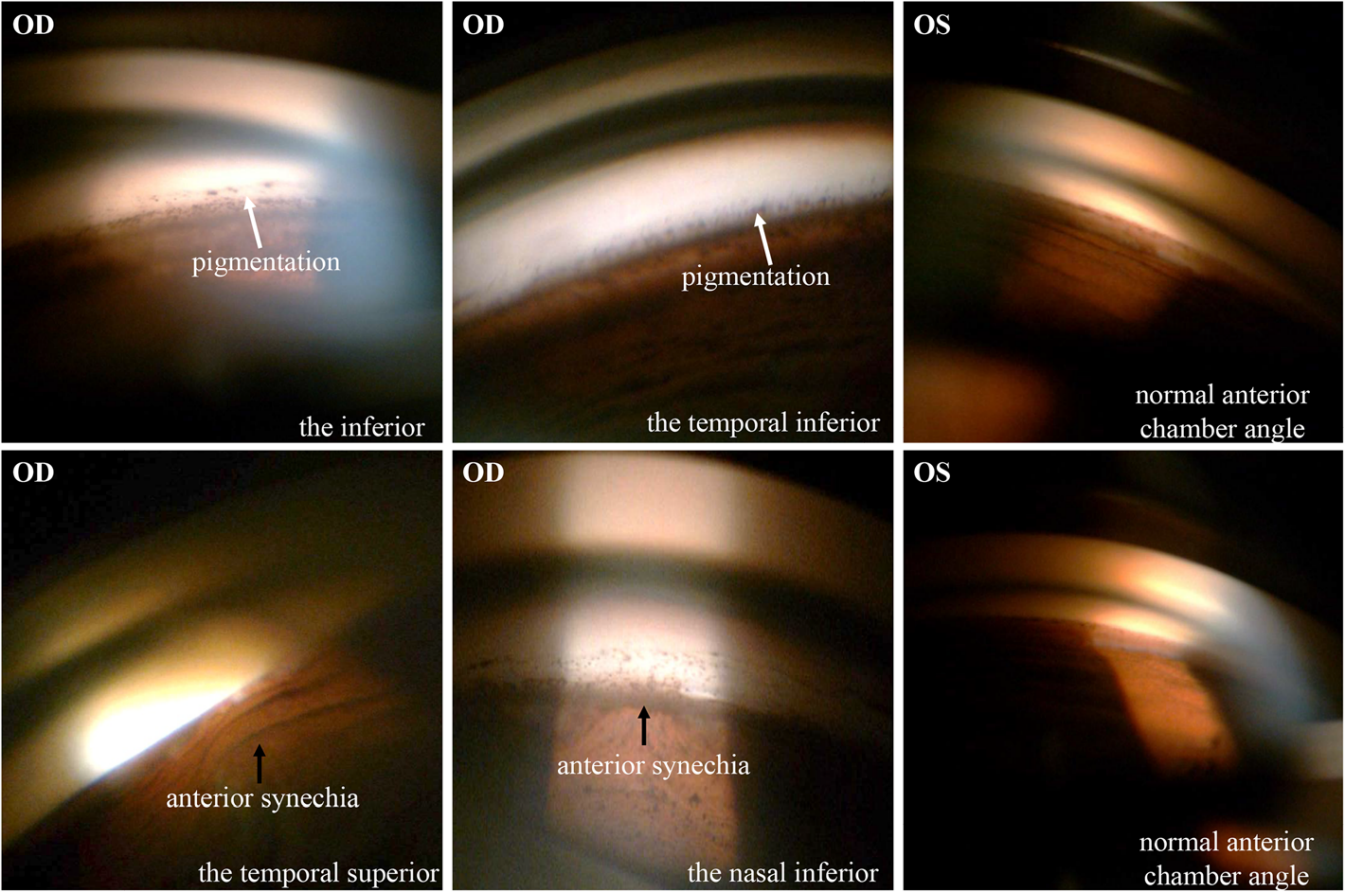
Gonioscopy. Massive pigmentation was detected in the chamber angle of the right eye (white arrows), along with anterior adhesion of the iris and angle closure in the superior temporal quadrant and the inferior nasal quadrant (black arrows). The chamber angle in the left eye was normal

**Fig. 4:**
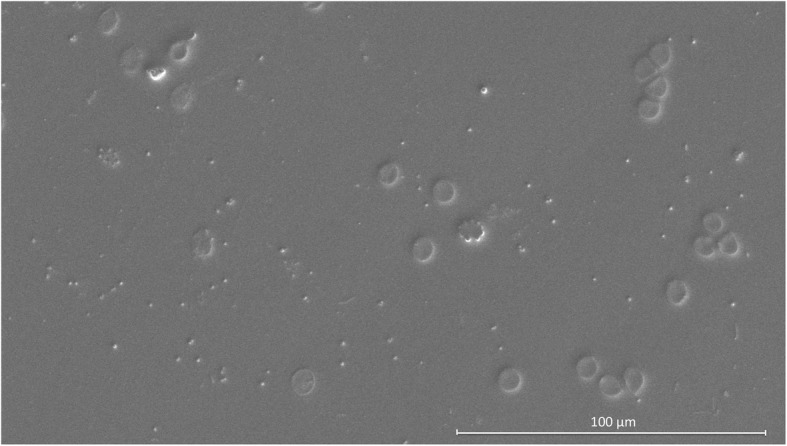
Electron microscopic analysis of the explanted intraocular lens. The microscopic analysis found no evidence of bacteria but some pigment cells and blood cells were present on the intraocular lens

She finally came to our hospital in September 2017. After careful review of her medical history, a series of detailed ophthalmic examinations were conducted. A slit-lamp examination revealed depigmentation and atrophy of the iris, and a quiet anterior chamber in the right eye. Fundus photography showed an opaque image of the posterior segment (Fig. 1a). B-scan ultrasound showed a mass of echoes throughout the vitreous cavity and strip-like echoes in the peripheral region (Fig. 1b). Optical coherence tomography revealed a retinal structure that is common in highly myopic eyes (Fig. 1c). The perimetry of the central visual field was normal without defects typical of glaucomatous eyes. Several conventional diagnoses were first considered, including infectious endophthalmitis caused by hypovirulent bacteria, noninfectious uveitis, and secondary glaucoma. However, these did not fully explain the combination of symptoms or the recurrent onset of the syndrome. Considering the features at onset, a possible diagnosis of UGH syndrome was proposed. Ultrasound biomicroscopy (UBM) was conducted with careful inspection of the chamber angle in all directions, and detected high echoes in the sulcus at the 5 and 11 o’clock positions, which was the direction in which the haptics of the IOL were placed. The root of the iris was also pushed forward in the same positions (Fig. 2). Gonioscopy revealed massive pigmentation in the chamber angle of the right eye and anterior synechia and angle closure, whereas the chamber angle in the left eye was normal (Fig. 3). Chafing of the posterior iris by the IOL haptic almost confirmed the diagnosis of UGH syndrome, although it was still necessary to exclude delayed endophthalmitis. IOL explantation and anterior vitrectomy combined with an intraocular injection of antibiotic were performed in the right eye. The removed IOL was sent for bacterial culture and electron microscopic analysis, which revealed no bacteria but some pigment cells and blood cells on the IOL (Fig. 4). At the follow-up 1 month after surgery, the patient’s visual acuity was 0.8 with an ideal IOP of 13.3 mmHg, without IOP-lowering medications.

## Discussion and conclusions

UGH syndrome has been reported in patients with anterior-chamber IOLs, and mechanical irritation caused by malposition of the IOL is the underlying mechanism. In recent years, UGH syndrome has also been reported following the placement of a posterior-chamber IOL. In this case report, we present a rare case of UGH syndrome with multiple episodes of transient visual blurring, floaters, eye pain, redness, and increased IOP, arising from malposition of the haptic of a sclera-fixed two-haptic IOL in a highly myopic eye.

In 2003, Foroozan et al. [2] described a case of UGH syndrome after in-the-bag placement of a single-piece lens. Repeated examinations during the symptomatic episodes revealed microhyphema, consistent with red blood cells in the anterior chamber. UBM showed the proximity of the edge of the IOL optic to the inferior pupillary margin in the region of an iridociliary body cyst. This case was the first to show that UGH syndrome might occur despite intracapsular placement of a posterior-chamber IOL. Badakere et al. [3] also described a case of UGH syndrome with a seemingly well-placed posterior-chamber IOL. Dilated gonioscopy was very helpful in that case, providing direct evidence of hyphema in the inferior capsular bag. The cause was noted to be a haptic displaced through a tear in the equatorial part of the bag. In 2014, Zhang et al. [4] proposed two additional mechanisms of UGH syndrome in two patients. In one patient, the zonular laxity from pseudoexfoliation syndrome caused pseudophacodonesis, leading to chafing of the posterior iris by the square-edged haptic. A capsular tension ring was implanted to redistribute the zonular tension and reduced the symptoms. In the second patient, the anteriorly rotated ciliary processes in the plateau iris configuration and focal capsular fibrosis around the haptics caused chafing at several points. Focal endoscopic cyclophotocoagulation of the affected ciliary processes was performed to retract them from the area of haptic impingement, and the UGH syndrome was treated.

Unlike these previously reported eyes with UGH syndrome after in-the-bag implantation of an IOL, our patient had a sclera-fixed two-haptic IOL and a history of multiple previous eye surgeries. At the time of presentation, these had caused multiple tissue lesions and made the structure fragile. Because the eye was also highly myopic, the stability of the capsular bag and the support it provided to the IOL were further impaired, which might have been responsible for the initial displacement of the IOL.

In the first of several episodes of blurred vision, floaters, eye pain, and redness, the patient was only given symptomatic treatment to relieve the inflammation and reduce her IOP, with no clear diagnosis of the etiology or pathogenesis of her eye disease. This temporarily mitigated the complaint, but recurrent episodes followed. Delayed-onset postoperative endophthalmitis was first considered when the patient attended our hospital, based on the typical complaints of blurred vision, redness, and pain. Reduced media clarity and poor fundus visualization are among the major clinical signs of endophthalmitis [5]. The recurrent episodes and remission after medication also suggested infection with hypovirulent bacteria. However, this did not fully explain the elevated IOP and bleeding. Although rare, UGH syndrome produces symptoms and signs that were observed in our patient. Under such circumstance, gonioscopy and particularly UBM allow the direct evaluation of the anterior chamber and the chamber angle, and provide essential clues to clarify the etiology [6]. In this case, the identification of mechanical chaffing of the iris by the malpositioned IOL supported the diagnosis of UGH syndrome, and greatly assisted the doctor’s selection of a therapeutic strategy. IOL explantation successfully eliminated the cause of chafing and the symptoms did not recur. Bacterial culture of the IOL was negative, excluding the possibility of endophthalmitis. The electron microscopic findings for the explanted IOL were similar to those reported by Asaria et al. [7] in a patient with UGH syndrome, further supporting the diagnosis of UGH syndrome in this case.

The necessity of primary IOL placement by sclera fixation after IOL displacement must be reconsidered. The eye was highly myopic, and according to the IOLMaster report, a 0D IOL would be used if targeting for plano after surgery. Repeated surgery and implantation and explantation of the IOL are risk factors for UGH syndrome. Doctors should be cautious in making this decision in such circumstances, considering the multiple tissue lesions induced by previous surgery and the invasive manipulation involved in IOL scleral fixation. Therefore, at our hospital, the eye was managed by removing the cause of the mechanical irritation of the iris (i.e. the IOL) only, which yielded satisfactory clinical outcomes in terms of the recovery of vision and IOP control. Although transscleral fixation of the IOL is safe and usually has favorable visual outcomes in aphakic eyes [8], doctors should be careful in the selection of the IOL for highly myopic eyes because the eyeballs of these patients are larger compared with emmetropic eyes.

Eyes with UGH syndrome can be managed with topical IOP-lowering steroids or other anti-inflammatory drugs. However, the symptoms might not be completely resolved if there is no clear diagnosis and the mechanical stimulation persists. In most cases, surgical intervention may ultimately be necessary [1].

In summary, doctors should be cautious in the choice of sclera-fixed IOL in patients with a history of multiple eye operations. It is important to use gonioscopy, together with UBM, to identify evidence of iris irritation in UGH syndrome and to understand its possible etiology. A differential diagnosis of postoperative endophthalmitis is also necessary.

## Data Availability

The datasets used and/or analyzed during this study are available from the corresponding author on reasonable request.

## Abbreviations

IOL: Intraocular lens
IOP: Intraocular pressure
UBM: Ultrasound biomicroscopy
UGH: Uveitis–glaucoma–hyphema
